# How the pandemic has affected Turkish housing affordability: why the housing running cost is so important

**DOI:** 10.1186/s40410-021-00132-3

**Published:** 2021-05-12

**Authors:** Seyda Emekci

**Affiliations:** Department of Architecture, Ankara Yıldırım Beyazıt University Esenboğa Külliyesi Dumlupınar Mahallesi Esenboğa, Ankara, Turkey

**Keywords:** COVID-19, Pandemic, Housing affordability, Housing running cost, Turkey

## Abstract

The housing affordability problem in Turkey is not new. With the pandemic increasing pressure on the economy, the issue of housing affordability problem has reached an alarming level. The problem has been deepened not only as a result of the pandemic but also due to the incomplete and wrong policies from the past. This paper on the one hand aims to examine how the pandemic has exacerbated the problem; on the other hand, it purposes to reveal that the problem has been handled incorrectly and how weaknesses in the policy strategies contribute to this problem through a case study of the low-income group. The article also focuses on how architects can contribute to solving this problem.

## Introduction

The COVID-19 pandemic has resulted in thousands of deaths and infected hundreds of thousands more (JHU 2021). Governments have put some measures in place to try to minimize the number of infected people from the disease. Since keeping mortality as low as possible, several nations have been applied lockdown restrictions. As a result of this, the COVID-19 pandemic is not only a health crisis but rapidly becoming an economic one too. According to the Work Bank data, each region experienced economic contractions, with Latin America by 7.2%, Europe and Central Asia by 4.7%, the Middle East and North Africa by 4.2%, Sub-Saharan Africa by 2.8%, and South Asia contracting by 2.7% (World Bank 2020). As many countries are experiencing a recession, the historic increase in joblessness has been bought on (World Bank 2021). For example, the United States, the largest economy in the world, rapidly rose to 14.7% in April following the impact of the Pandemic, while the unemployment rate was 3.5% at the end of February in 2020 (U.S. Bureau of Labor Statistics Data 2021). Although the devastating effects of the pandemic on the economy have also been felt in Turkey, the situation was different in Turkey. In Turkey, even before the pandemic, the unemployment rate was reported in the May of 2019 as 12.8 (TUİK 2019), with the addition of the pandemic to the already bad economic situation, it reached 13.4 in the july of 2020 (TUİK 2020) despite of the fact that work termination has been banned for a period of three months in Turkey and labor force participation rate fell. Along with unemployment at historic highs, the housing crisis in the country, which has not been comprehensively addressed before, is getting worse.

Construction has been one of the major sectors of the Turkish economy. Housing investment or the value of new housing construction plus the net addition to existing housing stock has comprised 9% of GDP on average since 2010 (TUİK 2020). Turkey has produced significantly high housing units since the year 2002, with the considerable increase of the authorities given to the Housing Development Administration of Turkey (TOKİ), the top official agency responsible for producing housing in Turkey (TOKI 2019). Although Turkey has produced a high number of housings since the year 2002 when public and private housing production was triggered by the government (Türel and Koç 2015), house prices continue to increase (both for the existing and new). According to data provided from The Central Bank of the Republic of Turkey (TCMB), as a result of the comparison of the Residential Property Price Indices (RPPI) of 2010 (the first year of this data) and 2020, it shows that both the index value and the square meter unit prices have tripled across the country (TCMB 2020). This significant increase in housing prices made access to affordable housing even more difficult. Especially low-income groups have been experiencing great levels of hardship regarding reaching affordable housing stemming from this increase. In Turkey, affordable housing has been offered only by the Housing Development Administration of Turkey for a low-income group called “social housing”. Besides the “social housing” is not sufficient in number, they are not affordable when considering their life cycle costs (Emekci̇ 2018). In addition to that, there are no subsidies for low-income tenants (Türel and Koç 2015). Due to the lack of state support, and the incorrect and incomplete handling of the concept of affordability, lower-income families are extremely vulnerable to changing market conditions and increasing the price of housing in Turkey. The affordability problem that has been already existing is exacerbated by the pandemic. It contributed to this problem by rising inequality, unemployment rate, and housing insecurity. The study aims, on the one hand, to analyze how the pandemic deepens the affordability problem that has been already existing in Turkey; on the other hand, to depict how the architect can play a role in the solving of this problem through by discussing on the low-income group. These purposes require, to clarify what affordability is, how it is measured in international and national literature at the very first stage, and to discuss especially in Turkey how the problem has been addressed so far and how the problem should be addressed.

## Definition and measurement of the housing affordability

Since the term is associated with multiple issues (i.e., housing quality, housing condition, housing costs, household income) and people who have different roles (i.e., tenant, owner), it is impossible to define housing affordability in a simple way. Similarly, a wide variety of methodological approaches has been used to measure it (Mattingly and Morrissey 2014). Although there has been a growing scholarly concern for this problem, there is no agreed definition and measurement method of housing affordability in the literature (Li 2014). However, it is possible to describe housing affordability in terms of the relationship between housing expenditures (prices, mortgage payments, or rent) and household income (Stone 2006; Thomas and Hall 2016). Similarly, Maclennan and Williams (1990) also pointed out this relationship, just as Hulchanski (1995) described the problem as *“pays more than a certain percentage of its income to obtain adequate and appropriate housing”.* While Hancock (1993) supports this relationship, she shifted in discourse from housing affordability to housing expenditures. She emphasized that *“the essence of the concept of affordability”* (p. 129) is the housing expenditures by discussing “*what income to be foregone in order to obtain housing and whether that which is foregone is reasonable or excessive in some sense”* (Hancock 1993, p. 129). After that, housing expenditures have been discussed as implicit cost in the concept of affordability (Stone 2006; Thalmann 2003) It contains tips for dealing with this problem correctly. Households can make a trade-off between their housing consumption and non-housing consumption. In other words, they may choose to spend a large portion of their expenses on housing expenditures and make the housing *"affordable"*. But where they do not choose to spend, it leaves them below the norm.

As there are differences in the definition of housing affordability, there is no consensus on how to measure it. Two of them that dominate the literature are as follows.

- The Ratio Approach advanced by Bogdon and Can (1997), Hulchanski 1995; O’Dell, et al. (2015); Wilcox (1999), Yates (2016) and Been et al. (2019)
- The Residual Income Approach, employed by Grigsby and Rosenberg (1975), Malpass and Murie 1999, Stone (1993), Kutty (2005) (Leishman and Rowley 2012), and Padley and Marshall (2019).

The ratio approach is the measurement of the housing affordability that means the ratio of the housing cost-to-income ratio, as defined 30/100, as stated by Kutty (2005) over time the thresholds *“have been set at 25 percent, 30 percent, 40 percent, and 50 percent”* (p. 115). Although the approach has been widely used because of easy to calculate, it has been mostly criticized due to the arbitrary threshold levels and the exclusion of non-housing consumption and housing quality. The residual income approach is the net income that refers to the income remaining for housing expenditures after subtracting the non-housing costs of families (Dolbeare 1966). Although it offers some solution to the problem associated with the ratio approach, there is no clear information about what an acceptable housing standard and housing expenditures are. Literature could not even agree on “what is an acceptable housing standard” because the concept is so vague and relative (Edgar et al. 2002), it is understandable that there is no clear definition of housing expenditures.

Housing expenditures, on the one hand, are described as only housing cost that is conventionally based upon the mortgage repayment in many countries (Bogdon and Can 1997; Linneman and Megbolugbe 1992); on the other hand, it is not based on independent cost information (Gabriel et al. 2005; Norris and Shiels 2007) that ignores some important parameters that affect housing expenditures (i.e., the household size variety, household consumption patterns). The incomplete and incorrect handling of this term leads to a “broad-brush” calculation that is far from measuring the real cost (total cost) of the homeownership.

## Housing affordability in turkey: the problem from the past

The global financial crisis that emerged in 2008 affected the housing policy of countries. They shifted their policies to be more liberal (Hegedüs and Horvarth 2015) as international literature has increased (Li 2014). However, the process has not proceeded in this way in Turkey. Despite the negative economic climate affecting Turkey like other countries, it could neither has increased the interest of researchers in the affordability literature nor have significantly changed Turkish housing policy (Özdemir Sarı and Aksoy Khurami 2018).

### Key housing policy initiatives

In Turkey, the affordability housing debate dates back to the early years of the republic. With the proclamation of the republic, a new era has begun. As the regime changed, some regulations related to housing and urbanization were put in place. Some precautions were taken to solve the housing affordability problem since then. In the early years of the republic, Emlak Eytam Bank was established to construct low-cost dwellings and provide housing credit. In 1946 the bank was transferred to Real Estate and Credit Bank in order to expand its activities. Through the restructuring, it was aimed to find the solution to the government's housing finance problem (Keleş, 1990). WWII impeded searching for a solution to the housing affordability problem although Turkey did not take part in it. In the following years, together with the high rate of urbanization, people started to solve their problems in their own way and squatter settlements, named “gecekondu” emerged. In the years between 1963 and 1967, the government prepared the First Development Plan of the country for a five-year time. The Squatter housing law, enacted within the scope of this plan, aimed at preventing “gecekondulaşma” by supporting housing cooperatives. Similar approaches continued during the Second Five Year Development Plan (1968–1972). During this period, the Land Office was established for developing and producing land for social benefit activities and Cooperatives Act was enacted to regulate cooperative institutions. In the 1980s, the First Mass Housing Law and The Second Mass Housing Law were put into effect. As the former one provided housing credit through housing cooperatives without making any differentiation between households, together with the latter law, the issues of financial incentives, credits with low-interest rates, and other encouragement facilities (i.e. land provision, eased planning procedures) were regulated. After enacted these laws and the establishment of the Housing Development Administration (TOKI), social housing projects accelerated. Together with changing the role of TOKI in 1990, in the first decade of the millennium, the direct and indirect intervention of TOKİ to the housing market have been affected housing production and affordability on a country-wide scale. Before that, the measures taken had a limited effect on housing affordability.

After the 2002 country-wide election, the construction sector gained more importance than ever before. In 2003, the housing program for the low-income group and redevelopment of squatter housing sites defined under the “housing development and planned urbanization” objective in the Emergency Action Plan (UAP) (UAP 2003), had a direct impact on housing affordability. Since then, through several measures (i.e. The deregulation and liberalization of the legal and institutional framework (Balaban 2008), reduced VAT rates (Türel and Koç 2015)), the government has supported the private sector. However, for new construction produced by the private sector, the target group was the middle to highest income group, not the low-income group.

### Weaknesses in the policy strategies

Since the proclamation of the Turkish republic, some important measures were taken to handle the housing affordability problem. It is possible to divide the Turkish housing affordability problem process into two parts: pre-2002 and post 2002. Before the year 2002, the measures taken were not effective in the whole country. They were just like local initiatives. However, after 2002, some initiatives (i.e. Planned Urbanization and Housing Production Program Tenth National Development Plan KENTGES Integrated Urban Development Strategy and Action Plan) help solving this problem albeit limited. However, there were some certain problems related to the economy. While the problems regarding the economy (i.e. the budget deficit and inefficiency in the mortgage markets) weakened the power of the policies implemented, they encouraged the growth of squatter housing and luxury housing rather than social housing. The inability to develop an institutional form that could provide housing to the lower-income group and the non-theoretical formulation of the policies made the problem far from the solution (Stephen Ezennia and Hoskara 2019).

## How the pandemic has affected Turkey

The outbreak was triggered in December 2019 in China, then the virus continues to spread across the world, and it became pandemic. It has altered the socio-economic and health dimensions of many societies across the world. It has also affected Turkish socio-economic development negatively.

Since 2000, Turkey has shown impressive performance in economic and social development. The Gross Domestic Product (GDP), which was $201,753 in 2001, reached $957,799 in 2013 (World Bank 2014). Turkey started to become an upper-middle-income country (Yilmaz 2014). Government programs focused on vulnerable groups and disadvantaged areas. As the poverty headcount ratio at national poverty lines (% of the population) was 18.6 in 2005, the ratio reached 13.5 in 2016 (World Bank 2017). The poverty rate has declined by 5% between 2005 and 2015. After this date, due to increasing inflation and unemployment, shrinking investment, increasing institutional and financial sector vulnerabilities and irregular implementation of corrective policy actions and reforms, Turkey's macro-economic picture has become a vulnerable and uncertain (World Bank 2020). With the pandemic, the situation has worsened.

**Fig. 1:**
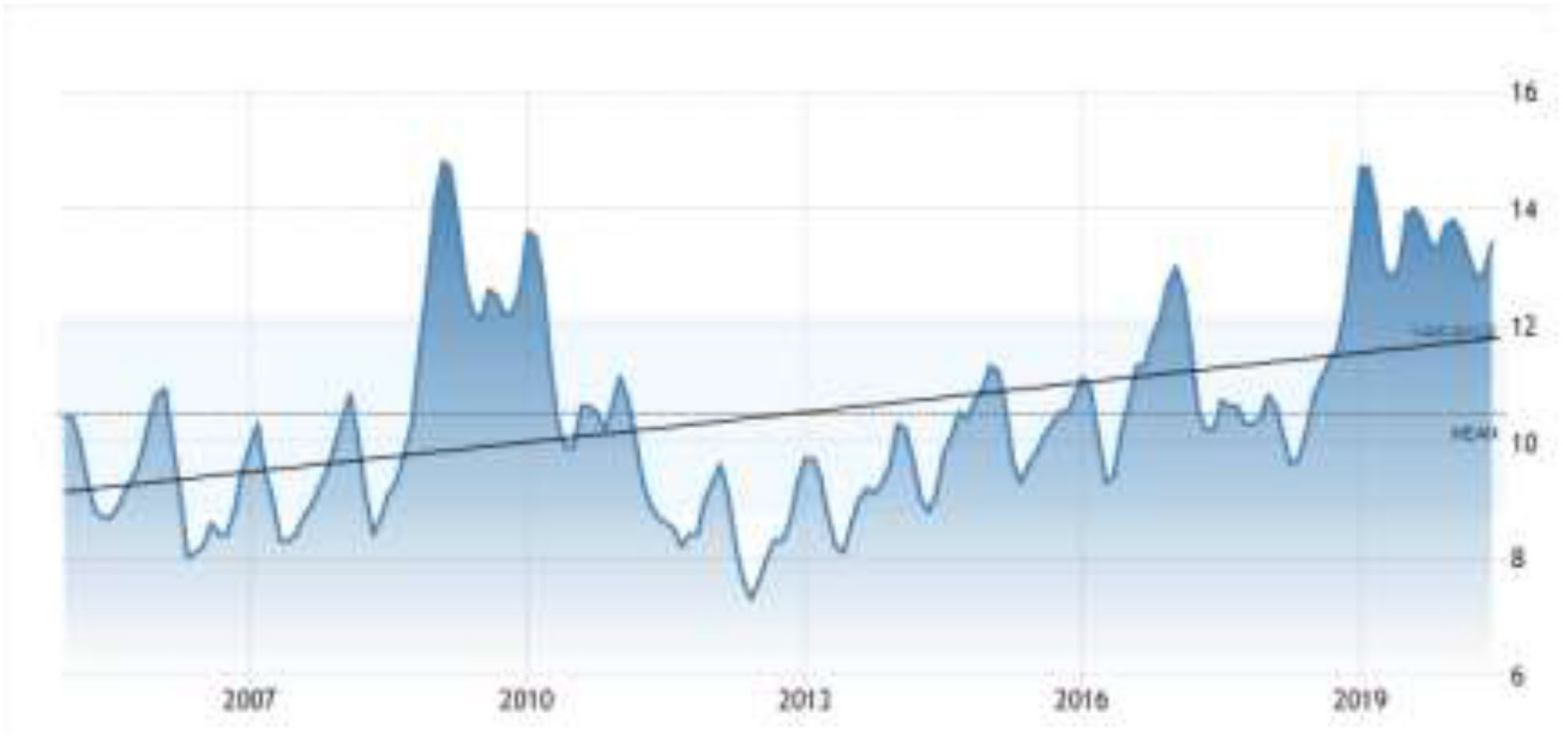
Changing of the unemployment rate over the years in Turkey

According to Turkish statistical data, the unemployment rate in Turkey averaged 10.45 percent from 2005 until 2020 (TUIK 2020). It has reached the highest level since February approximately when Turkey confirms the first case of coronavirus. Although the labor force participation rate fell from 53.3 percent in the same month last year to 49 percent, the unemployment rate is similar to the rates in the 2008 global economic crisis. The youth unemployment rate between the ages of 15–24 increased from 24.9 percent to 26.1 percent in May of 2020 (TUIK 2020). These rates clearly show that even before the effects of the pandemic on the country's economy are felt, unemployment is on the agenda of the country as an economic problem (Fig. 1).

**Fig. 2:**
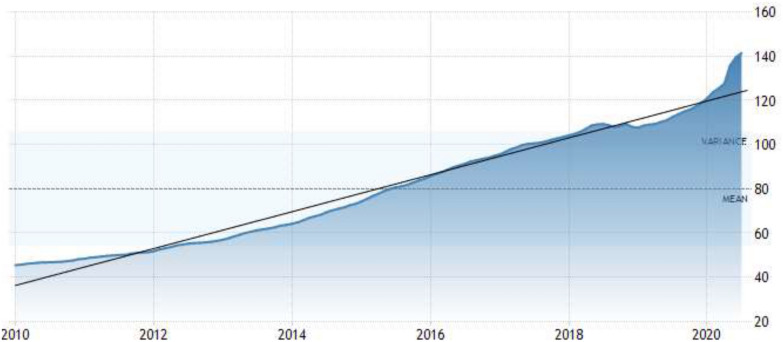
Changing of the country’s nationwide house price index

In the same period, parallel to the unemployment rate, the country’s nationwide house price index soared. According to the Central Bank of the Republic of Turkey (CBRT), the Housing Price Index in Turkey averaged 80.01 points from 2010 until 2020. It has been reaching the highest level at all time in July of 2020. Along with the COVID-19 pandemic hits Turkey, during the year to Q1 2020, as the price index for new dwellings soared by 18.77%, it for existing dwellings rose 14.25% (Fig. 2).

**Fig. 3:**
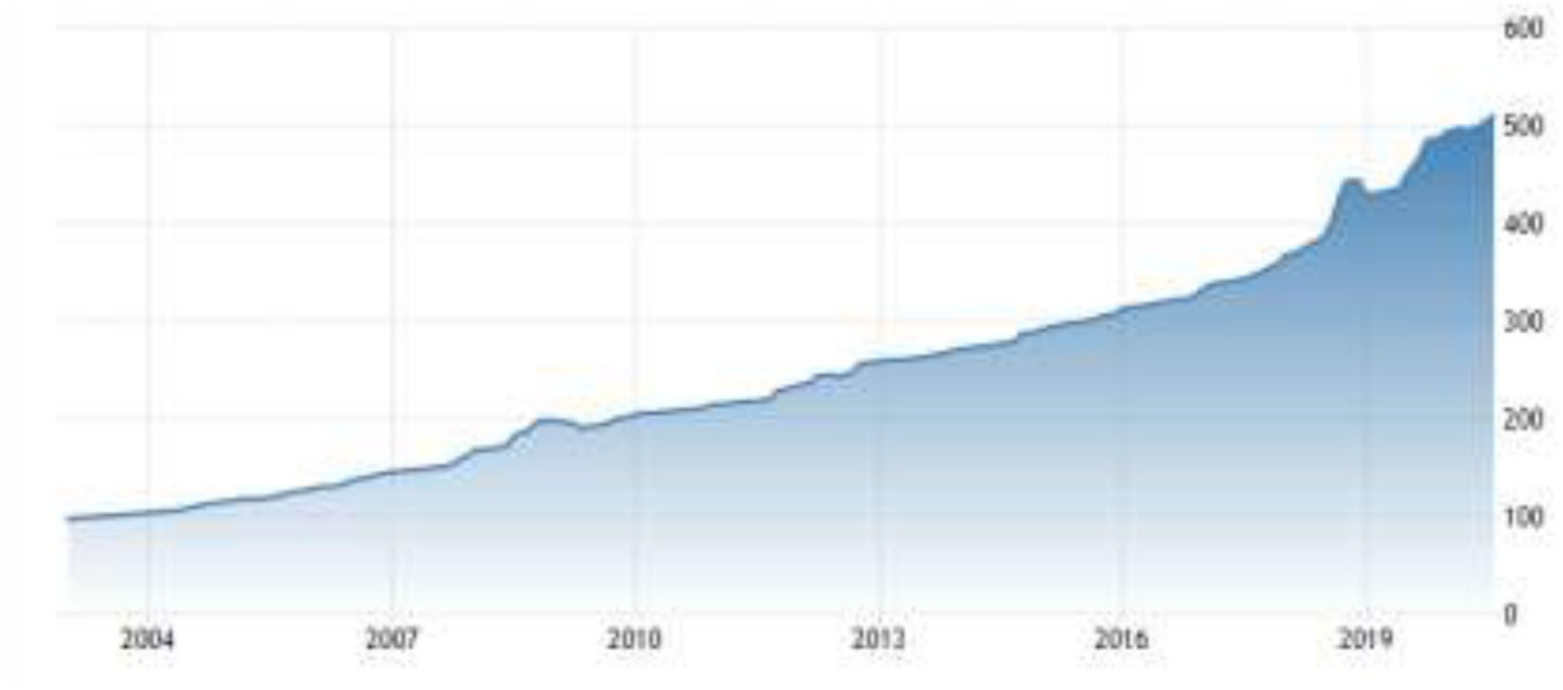
Changing of the CPI housing utilities

According to Turkey Statistical Institute (TUIK) the household budget survey data, “Housing Utilities “ has been one of the main burdens of households after the rent cost (TUİK 2020). The share of spending on housing utilities such as electricity, heating, etc. consists of 1/4 of household spending (TUIK 2020). In approximately the last two decades, it constantly soars (Fig. 3).

Despite declining energy prices due to the Coronavirus pandemic all over the world, in Turkey housing utilities including energy that has been used in residential buildings rose to 510.03 points in August from 505.21 points in July of 2020 with exchange rate pressures (IEA 2020).

## Methodology

This paper employs case studies, surveys, and in-depth interviews as a method to investigate and answer the research question. The case study method with the survey in the context of qualitative research constitutes the backbone of the study as in-depth interviews create the infrastructure of the paper. The survey was conducted with 150 households in Ankara TOKI Mamak Karakusunlar Housing produced for the low-income group which was determined as a case study area. The number of housing in the project is 4022. When the density calculation is made according to the household size of 3.8 for Ankara, the number of people in the case study area was calculated as 15,284. The sample size was determined as 73 people at a 95% confidence interval, using the formula below.

1 $${\text{n }} = {\text{ Z2 NPQ }}/{\text{ ND2 }} + {\text{ Z2 PQ}}$$

n = Sample size,

Z = Confidence coefficient (1.96).

P = Probability of the feature to be measured in the mass (95%) (0.95).

Q = 1-P (0.05).

N = Population size.

D = Accepted sampling error (5% sampling error was foreseen for the study) (0.05).

However, the survey was carried out with 150 people in order to obtain more reliable results from the study. The survey was conducted with an individual who can represent the household between June 5–20, 2020. By using different question types (multiple-choice, single and multiple-answer questions, and finding answers to questions not asked with the other option), it is aimed to reflect the participants' thoughts and experiences (Bryman 2008; Agresti and Finlay 2003). Participants have the freedom to not answer any questions and/or leave the research whenever they want. Before starting the survey, the purpose of the study and the participant's rights would be explained in detail to the participant. SPSS program could be used to analyze the collected data healthily and systematically (Bryman 2008; Creswell 2009). Another benefit of this analysis program is that it allows for in-depth analysis of any data without being lost or shared with anyone else (Creswell 2009; Mason 2006).

## Case study

As it confronts the COVID-19 pandemic, many countries face economic recession. Unemployment and income losses are common but more prevalent among low-income families (Karpman et al. 2020). Since the hardest-hit income group is assumed to be low-income families in Turkey as it is in other countries, the low-income group was selected as a case study. In this research, Ankara TOKI Mamak Karakusunlar Housing was examined. According to TOKİ, the selected target group has a maximum income of 3200 ₺ (TOKI 2016) or not having been covered by any one of the social security institutions (TOKI 2010). It also means higher inspiration for affordable housing solutions. Choosing a middle and high-cost building may prevent understanding of the deep impacts of the pandemic.

**Fig. 4:**
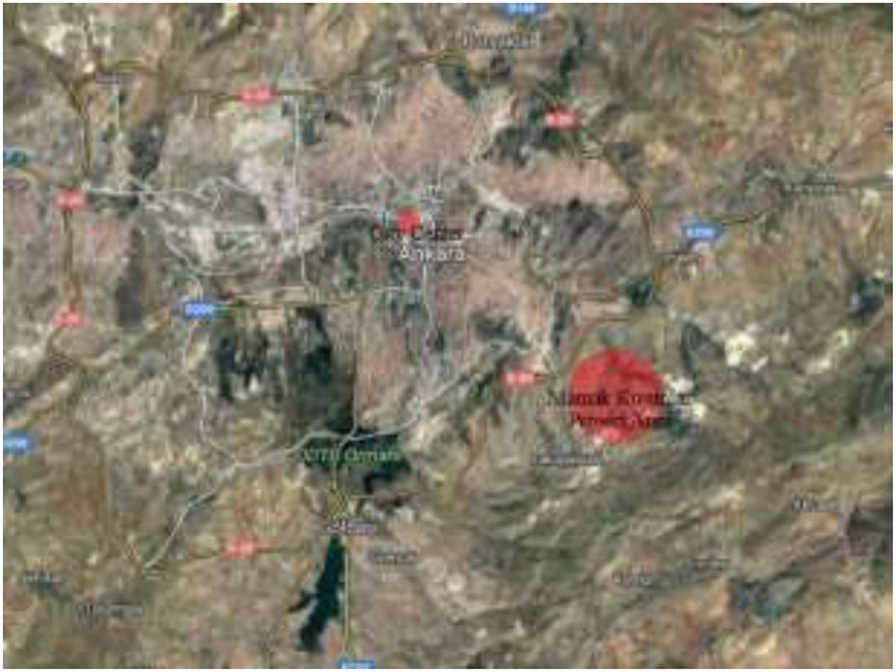
Site plan of the case study

The project is located at Mamak, Ankara, with a 17 km distance from the city center. There are no settlements in the vicinity of the Kusunlar Project Area (Fig. 4). The housing project has been completed by March 2014. This area has been used for squatter housing owners who are within the scope of urban transformation projects in different regions of Ankara. Ankara Mamak Kusunlar Urban Transformation Project consists of 4022 housing units. The buildings have 12, 13, or 14 stories.

## Results and discussion

The COVID-19 pandemic has put many people out of jobs by affecting countries’ economies negatively. There is no doubt that every segment of society has been affected by the pandemic. However, the pandemic hits low-income groups hardest. Turkey has experienced housing affordability as an unsolved problem. Since long before the pandemic, low-income individuals have been experiencing great levels of hardship regarding paying their housing expenditures Pandemic is bringing to light an existing housing crisis that the low-income group has experienced. The survey was carried out on 150 households who are living in Ankara TOKI Mamak Karakusunlar Housing and shows how the Covid-19 pandemic affects their (1) current situation of households, (2) expectations from the future.

### Current situation: the low-income group hits hardest by Pandemic

Pandemic has affected every segment of society, but it exacerbated hardships for the low-income group. Job losses, decreasing household income are forcing families to meet needs such as housing, food, and medical care.

**Fig. 5:**
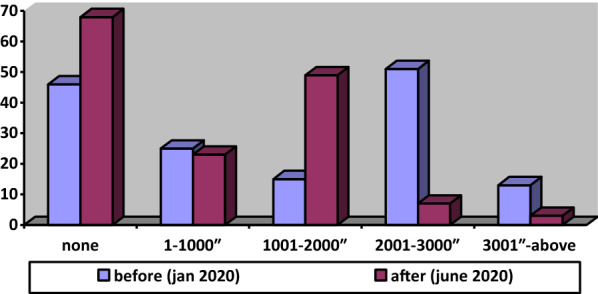
Distribution by household income level

As a result of the survey conducted in the case study area, the households in the sample group were grouped according to their sectors worked and a monthly income as indicated in Table 1 and Fig. 5, respectively.

**Table 1:** Distribution by sectors worked

Sectors	Percentage
Services Sector	74.6
Industrial Sector	18
Agriculture Sector	7.3

The Covid-19 pandemic and the associated lockdown measures have played a dramatic role in the sectors that rely on physically close human interactions. The services sector is one of them. According to UN Trade and Development Agency, the COVID-19 pandemic has hit hardest the services sector, especially tourism, hospitality, and retail (UNTDA 2020). According to Table 1, the majority of the people in the sample group work in the services sector with a rate of 75%.

When examining the figure, despite the increasing unemployment rate in Turkey, the number of people who lost their jobs has not increased at the same rate. Because according to Provisional Article 10 has been added on April 16, 2020, to Labor Law No. 4857, work termination has been banned for a period of three months. However, the employer may put the worker on unpaid leave, completely or partially. However, if an employee is given unpaid leave, for this reason, she/he can benefit from the state’s 39.24 TL daily wage support. That is why the number of people who belong to the group who earns 1001–2000 TL has been increasing at a high level.

**Fig. 6:**
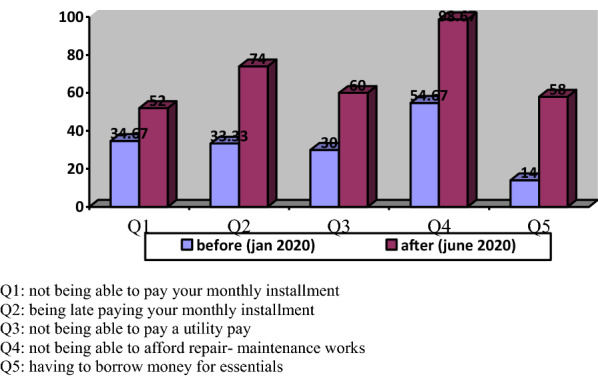
Percentages of households who have experienced economic difficulties

These results must be evaluated with the other parameters. As the minimum wage in Turkey has been determined as 2,558.40 Turkish liras per month, According to the research carried out by the confederation of Turkish Trade Union (TÜRK-İŞ), the monthly food expenditure known as the limit of hunger of a family composed of four has risen to 2.431,08 ₺ in June 2020 from 2.067,17 ₺ in June 2019. When compulsory expenditures (i.e. clothing, rent, electricity, water, and heating are added to the food expenditures), the limit of poverty increased 7.918,82 ₺ in June 2020 from 6.733,44 ₺ in June 2019. Even the monthly living cost of a single worker was calculated as 2.952,41 in June 2020. Before the pandemic, according to Fig. 6, the percentage of those with an income of 3000₺ and below is 91.3%. The percentage reached 98% after the pandemic. These numbers give information about how the low-income group in Turkey struggle to live before the pandemic and also show how the hardest pandemic hits them.

Turkish housing system has some differences from other developing countries’ one. Firstly, social housing has been produced only by the public sector through the Housing Development Administration (TOKİ) for low- and middle-income households in Turkey. TOKI produces flats for owner-occupation to households at subsidized rates. The private construction sector avoids involvement in social housing production due to commercial concerns. Secondly, most Turkish households do not rely on mortgages to purchase a house. Personal saving constitutes the majority source of capital that uses to buy a house. According to research carried out by TOKI (2006), 75% of homeowners who do not employ mortgage use their personal savings as financial sources to purchase a house. That implies households who get housing from TOKİ have no mortgage repayment, but they are indebted to TOKİ. Beneficiaries of social housing built on TOKİ's own lands make their down payments before they start to settle in their housing at the beginning of the construction period, and their monthly payments continue according to the repayment plans established by TOKI. Monthly installments for low-income groups are increased at the rate of whichever is the lowest among the indicators of the public sector wage index, domestic producer price index, or consumer price index (TOKI 2020). For instance, according to TUİK (2020), the indicators increased respectively by (public sector wage index) 5.49%, CPI (5.75%), and D-PPI (6.89%) in the previous six-month period (1 January 2020—30 June 2020). TOKI updates the monthly installment by considering the lowest of these rates. This ratio is 5.49%. Monthly installments are increasing twice a year according to the indicators given. This is only a payment resulting from the purchase of housing. Even when the other costs regarding the housing are not included, some households experience housing affordability problems as shown in Fig. 6.

In the affordability problem, housing affordability has been commonly described as short-run indicators that compare household income with housing costs. Despite the fact that some researchers emphasize the importance of housing expenditures (Abeysinghe and Gu, 2011; Özdemir Sarı and Aksoy Khurami 2018), in the literature the definition of the “housing expenditures” are still under discussion. In most cases, housing running costs overlook. Ignoring the existence of the housing running cost causes this problem to be handled incorrectly and delays finding an accurate solution to this problem. Running costs which cover utility payments, repair-maintenance costs, services, and property taxes have an important place in the budget compared to the initial cost of the housing (Mithraratne and Vale 2004; Pellegrini‐Masini et al. 2010; Wang et al. 2005; Wong et al. 2010). According to the literature, a typical household spends a considerable part of monthly income on housing utilities such as electricity, heating, and water (Fankhauser and Tepic 2007). Especially Turkish housing system affordability means “affordability of running costs associated with the dwelling” (Özdemir Sarı and Aksoy Khurami 2018). When analyzing the figure, as before the pandemic, the percentage of the group who cannot afford utility payments was 30%, after the outbreak, this ratio increased by 50 percent. Almost all of the households participating in the research cannot allocate a budget for repair maintenance works. The affordability problem that households already have experienced has deepened with the outbreak of the pandemic. Pandemic led households to be unable to cover their running costs.

**Fig. 7:**
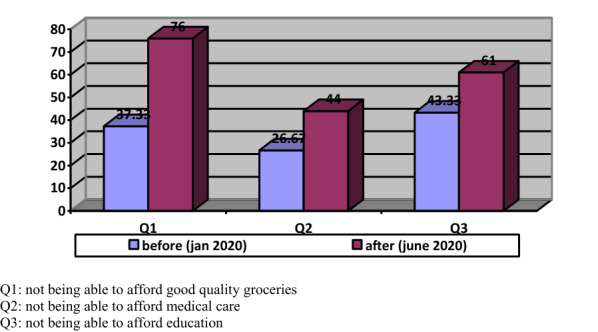
Percentages of households who unable to afford

The affordability problem is a multidimensional problem not only in terms of the issues it affects but also in terms of its effects. Since the household allocates an important share for the housing running cost that was never considered when owning -so-called- an affordable house, one would have to allocate less money for food, health care, and other expenses required to maintain a decent life as shown in Fig. 7.

The research found that 76% of households experienced unable to afford good quality groceries, while many of them are now facing education and health challenges. The findings come from the survey show that the pandemic has brought current inequalities to light.

### What will happen the next

**Fig. 8:**
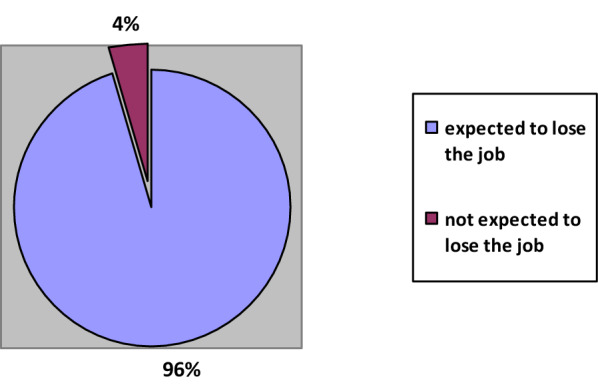
Percentages of households who expected to lose their job

The pandemic has affected not only the current situation of the households but also their expectations from the future. According to the research, households believe that the ongoing affordability crisis will only worsen in the coming months (Fig. 8).

Among those who are currently working, almost all believed that they would lose their job following the expiration of the Provisional Article 10 regarding that work termination has been banned for three months.

**Fig. 9:**
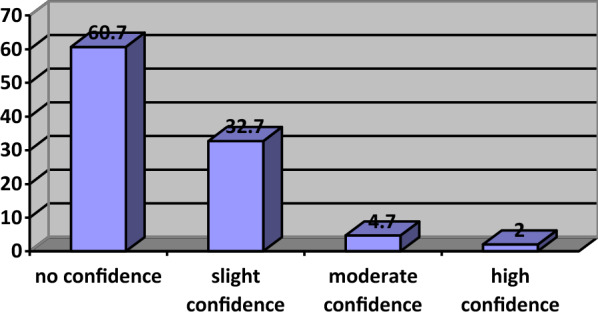
Confidence to pay household expenditures

Only 2% of households had a high degree of confidence they will cover housing expenditure (monthly installments, utility payments). Approximately 61% have no confidence or have already deferred monthly installment and utility payments (Fig. 9).

**Fig. 10:**
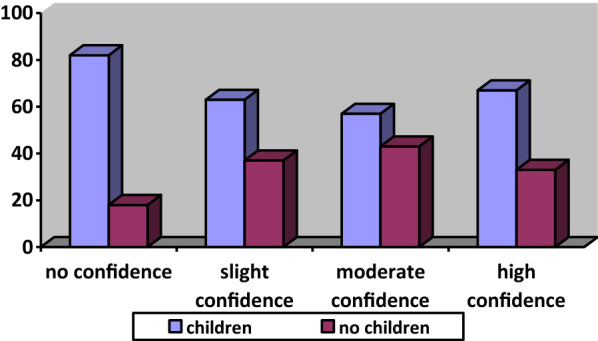
Confidence to pay next month’s installment in terms of having children

When another variable, “having a child” is added to the analysis, confidence drops sharply. These families have difficulty in paying the next month's installments, as other expenses (education, health, and food) of these families are higher than families without children. Confidence is also sharply lower amongst those who have children, reflecting an important socio-economic dynamic in housing ownership in Turkey (Fig. 10).

**Fig. 11:**
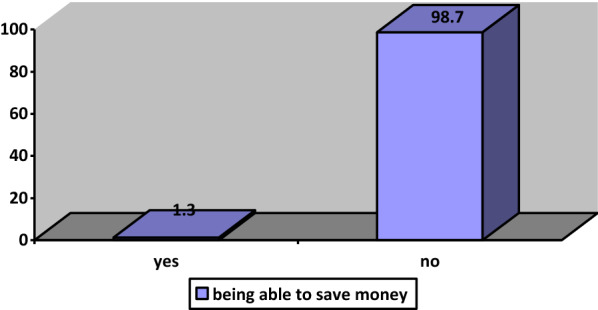
Saving money

Figure 11 states that only 1.3% save money to be used for unexpected situations. Since a significant majority does not cover their current expenses, they cannot make any savings (Fig. 11). According to the survey conducted by TUİK, 59.2% of those in the low-income group cannot afford their unexpected financing expenses, while this rate is just 9.9% for the high-income group (TUİK, 2020). This implies that these groups are particularly vulnerable to unexpected situations such as the Covid-19 pandemic.

## Solution: how the architect can help

The discussions, concerns, and views on this problem reflect the different assumptions and priorities of the different disciplinary groups. Each group deals with the problem from its own perspective. Similarly, the solution to the problem is specific to the different occupational groups. For example, as sociologists often focus on social inequality (Stone 1993), economists usually prioritize clarity of the problem (Quigley and Raphael 2004) and try to find the solution according to the problems they define. As the subject is housing, the problem should be extensively addressed by architects. The problem is far beyond the percentage of household income for the purchase price of a house. It is needed to tackle sustainably.

The literature on affordability has emphasized the threshold costs of accessing housing and on the ongoing mortgage repayment and rent payment. The costs have a direct influence on household consumption. They are predictable costs. That is to say, anyone can know how much they can pay before purchasing a property. However, being able to pay these costs does not make any housing affordable. Studies state that the running cost of housing has a significant share in the housing life cycle cost (Emekci̇ 2018; Mithraratne and Vale 2004; Pellegrini‐Masini et al. 2010; Wang et al. 2005; Wong et al. 2010). Since the costs do not calculate at the design phase, they tend to be ignored in the affordability debate. However, the housing running cost is vital to make housing affordable for especially low-income groups. Debating housing running costs is crucial especially at a time when economic shocks such as the Covid-19 pandemic have shaken all over the world. the costs make the low-income groups more vulnerable at such times.

According to the results of the research conducted within the scope of the paper, among the lower-income groups, since the vast majority of them work in the services sector most affected by the pandemic, the risk of losing their job at such times is very high. Every fluctuation in the economy makes them more vulnerable to housing expenditures. Accordingly, the group which already has a limited income experiences a significant decrease in their income. According to data get from the Policy Research Working Paper prepared by World Bank, there has been an inverse relationship between income level and the running cost budget share (Hope and Singh 1995). For instance, the higher-income households have a lower budget share for running costs as the lower-income households have a higher one. This implies that changes in the economic situation have affected the lower-income households mostly. The proportion of the housing running costs in their decreasing income has been gradually increasing. That is where architects can make a difference in determining whether housing is affordable.

**Fig. 12:**
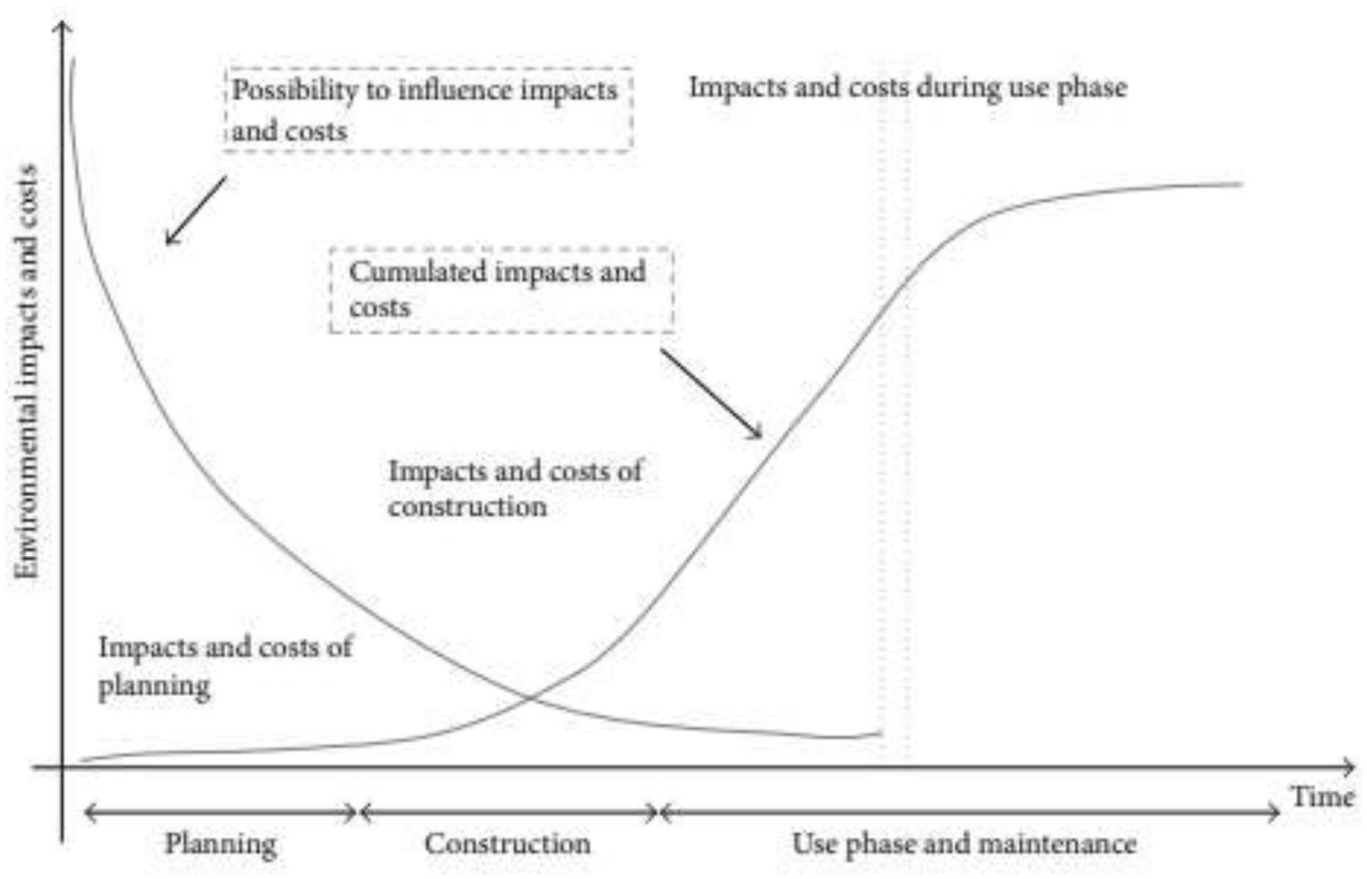
Influence of design decisions on life cycle impacts and costs. Source: Kohler and Moffat (2003)

To reduce the investment cost of housing, poorer quality inputs (i.e. construction methods, design and, choice of building materials) are generally used in housing produced for low-income groups (UNCHS 1995). This means that these groups actually have to pay more for living in their housing due to their high running costs. This fact brings the need for architects in the housing market, especially in the interpretation of housing inputs efficiently to the light. Decisions made in the early design phase of housing have a great impact on the running costs throughout its life (Bragança et al. 2014; Koukkari et al. 2005) (Fig. 12).

A project starts with the definition of its goals. In this first stage, the architect makes the decisions to develop the concept of the building, taking into account the characteristics of the target group.

The main purpose here is to produce solutions for the housing running cost, which are subject to the housing affordability literature, by making long-term and systematic analyzes at the early design stage. These suggestions should not only reduce the running costs in housing projects aimed at the low-income group, who are more vulnerable in times of economic shock but also do not increase the initial (investment) cost of that housing in a way that makes it difficult for this group to access housing. Architects can help by finding and implementing solutions that reduce running costs without increasing the investment cost of the housing. The architects can contribute through a building design that takes into account climatic characteristics. The climate in which the building is located in one of the main factors that play a role in the energy consumption of the building due to heating and cooling. Climate-sensitive buildings play a crucial role in reducing the energy demand of buildings without compromising modern living standards (Bodach et al. 2014). For example, in order to minimize the heating/cooling load of a building, many factors such as the surface-area-to-volume ratio, the orientation of the building with the main facades, the window area, the SHGC values ​​of the windows; insulation in the roof, colored outer surfaces, close building arrangements, mutual shading should be evaluated according to the climate in which the building is located in the planning stage (Mitterer et al. 2012). They also offer a solution through the choice of building materials. In material selection, it should be ensured that the material is reusable, recyclable, long-lasting, durable, not requiring frequent maintenance and repair, and the local material that requires less cost during extraction, processing, production, and transportation of the material from its source. In terms of heat loss and gain, high R-value material should be used in the building envelope (Farhat et al. 2014; Froeschle 1999; Spiegel and Meadows 2010).

By making long-term, systematic analysis, they can identify building hot spots that make up the running cost in the early design stage and find a solution to eliminate them. So, they make it possible to make more affordable and sustainable housing in terms of the housing running cost. The process must be done at the design stage. It can be decided whether the house is affordable before it is built. Some decisions can be taken, and changes can be made to make it more affordable. Even if they are impossible to make a house affordable, the occupant would be informed in terms of the effectiveness of existing low-cost housing projects.

## Conclusion

Turkey has made some attempts to deal with the housing affordability problem. Some of those were not effective in the whole country. They were just like local initiatives. Some initiatives have helped to solve this problem albeit limited. Despite the fact that all measures that have been taken so far provided temporary relief, none of them bought a long-term solution. With the emergence of the Covid-19 pandemic, the unsolved problem has deepened. The purpose of this article is to demonstrate how the housing affordability problem, which is still a problem today due to incomplete and wrong policies from the past, has deepened with the pandemic. In order to show that the low-income group, which is more vulnerable in times of economic shocks, was selected as the sample group.

The findings obtained as a result of the research have a number of implications. First of all, the pandemic underlined that although strategies implemented in Turkey in order to find the solution to the housing affordability problem have provided limited benefit, they could not bring a long-term solution. Since the problem contains economy-based issues, it is impossible to handle it independently of the country’s macroeconomic framework. Turkey’s macro-economic framework and its approach to the problem have negatively affected this problem. With the outbreak of the pandemic, the problem gets deeper.

Secondly, economic shocks such as the Covid-19 pandemic have a great impact on low-income groups because of their vulnerability to housing running cost problems. It is impossible to produce a strong solution to the problem without realizing what the housing affordability problem actually is. The housing affordability problem in Turkey has not been related to only housing purchase prices. The low-income households have allocated a significant share of the housing running cost. Not being able to find a systematic, long-term solution to this problem makes especially the low-income households more vulnerable to such shocks.

Thirdly allocating important share for the running cost has affected not only their economies but also socio-economic status. Because of this problem they must spare less money for food, health care, and other expenses required to maintain a decent life.

In order to reduce the effects of possible shocks such as the Covid-19 pandemic and to make the low-income group more resistant to such shocks, the architect can contribute to the solution of the problem by producing sustainable solutions that reduce running costs.

## Data Availability

Not applicable.
